# Supplementary material for: FGFR2 is essential for salivary gland duct homeostasis and MAPK-dependent seromucous acinar cell differentiation
Source: Nat Commun. 2023 Oct 14;14:6485. doi: 10.1038/s41467-023-42243-0 (PMC10576811; doi:10.1038/s41467-023-42243-0)
Supplement: Supplementary file 1 — Supplementary Information [file 41467_2023_42243_MOESM1_ESM.pdf]

# Supplementary Figure 1

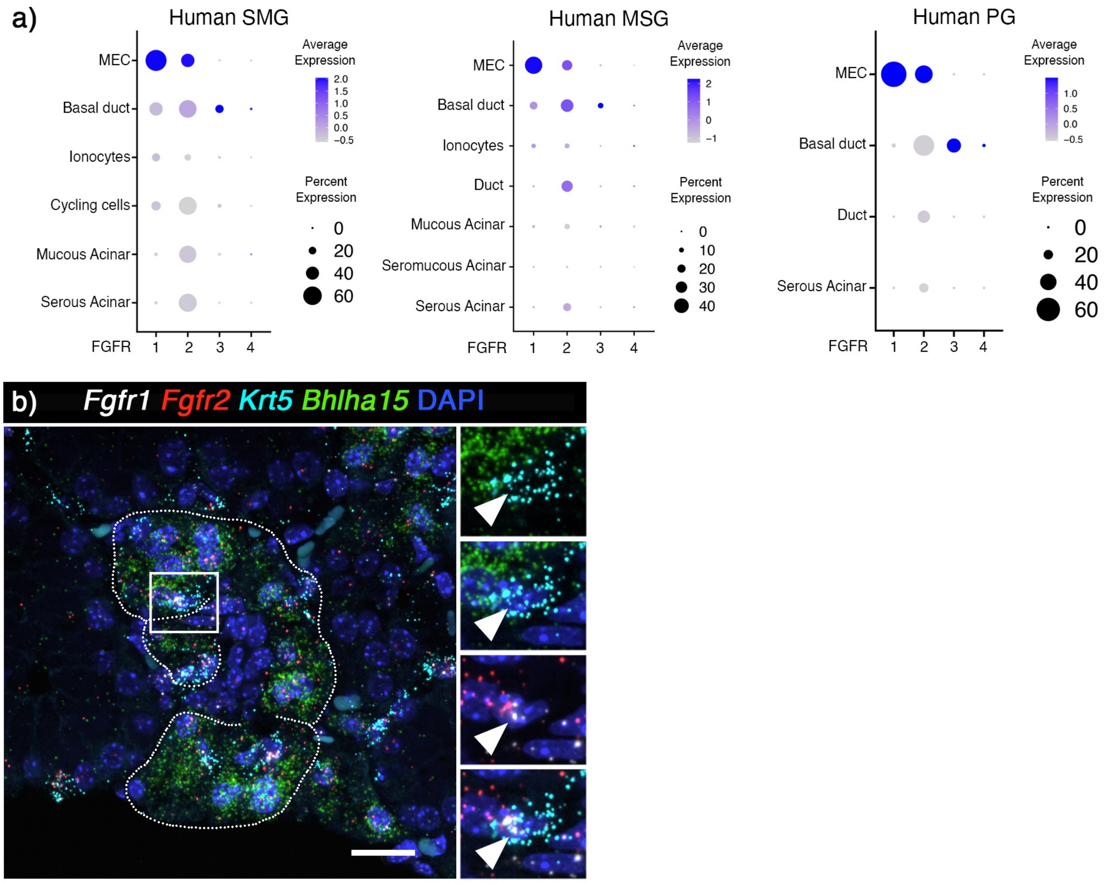


**Supplementary Figure 1: Postnatal human salivary glands have a similar expression pattern of FGFRs as mouse and *Fgfr1* and *2* expression are enriched in mouse MECs, related to Figure 1.**

a) FGFR expression in postnatal human salivary glands show similar overall pattern as found in postnatal mouse SMG. Enrichment of FGFR1/2 was found in Basal duct, MECs and acinar cells. FGFR3 was detected in basal cells of human salivary glands.

b) In situ hybridization with *Fgfr1* (white), *Fgfr2* (red), *Krt5* (cyan) and *Bhlha15* (green) showing enrichment of *Fgfrs* in MECs. Inserts show a *Krt5+* MEC adjacent to a *Bhlha15+* acinar cells expressing both *Fgfr1* and *Fgfr2*. Representative image from P30 SMG (n=3). Scale bar = 20 $\mu$m.

## Supplementary Figure 2

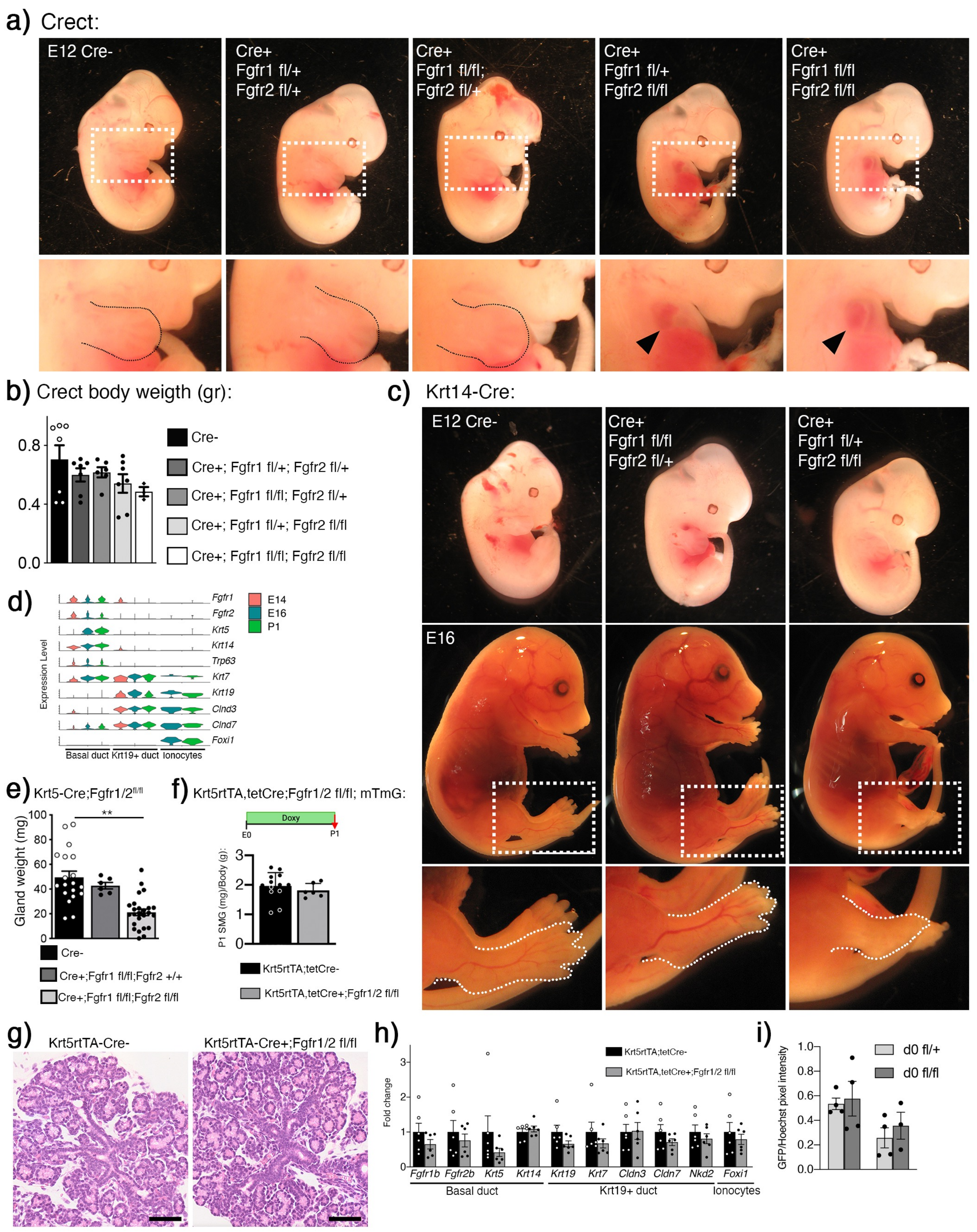

**Supplementary Figure 2: *Fgfr2* is required for normal limb and craniofacial development and salivary gland growth, related to Figure 2 and 3.**

a) While *Fgfr2*$^{fl/+}$ embryos are grossly normal and indistinguishable from *Crect-* littermates, *Fgfr2*$^{fl/fl}$ embryos fail to develop limbs (inserts and arrow heads). The phenotype observed in the *Crect+;Fgfr1*$^{fl/+}$*;Fgfr2*$^{fl/fl}$ embryos is exacerbated upon the loss of an additional *Fgfr1* allele. Representative images shown.

b) Although the mice displayed a dramatic phenotype, the average weight was similar in the *Crect+;Fgfr1*$^{fl/fl}$*;Fgfr2*$^{fl/fl}$ compared to the other groups. Unpaired two-tailed student t-test was used to calculate significance. Data is presented as mean values +/- SEM. *Cre-* n=7, *Cre+;Fgfr1*$^{fl/+}$*;Fgfr2*$^{fl/+}$ n=7, *Cre+;Fgfr1*$^{fl/fl}$*;Fgfr2*$^{fl/+}$ n=6, *Cre+;Fgfr1*$^{fl/+}$*;Fgfr2*$^{fl/fl}$ n=7, *Cre+;Fgfr1*$^{fl/fl}$*;Fgfr2*$^{fl/fl}$ n=3. Source data are provided as a Source Data file.

c) Gross images of *K14Cre+; Fgfr1/2fl/fl* embryos at E12 and E16. Deletion of *Fgfr2* in Krt14 lineage resulted in defects in digitization of the hind limbs (bottom, inserts). Representative images shown.

d) Duct populations from E14, E16 and P1 was bioinformatically isolated an integrated. Violin plots of scRNAseq data showing enrichment of *Fgfr1b* and *Fgfr2b* in basal cells compared to other duct cells.

e) SMG glands from adult *Krt5Cre+;Fgfr1*$^{fl/fl}$*;Fgfr2*$^{fl/fl}$ (n=24) were smaller than WT control (n=19) and *Krt5Cre+;Fgfr1*$^{fl/fl}$ mice (n=6). One-way ANOVA with Dunnett's test for multiple comparisons was used to calculate significance, **p<0.0001. Data is presented as mean values +/- SEM and source data are provided as a Source Data file.

f) Mice were fed doxycycline during pregnancy, and glands were isolated from P1 pups. Deletion of *Fgfr1* and *Fgfr2* in Krt5+ lineage did not affect gland size (Cre- n=13, Krt5rtTA;tetCre:Fgfr1$^{fl/fl}$;Fgfr2$^{fl/fl}$ n=6). Unpaired two-tailed student t-test was used to calculate significance. Data is presented as mean values +/- SEM and source data are provided as a Source Data file.

g) SMG from Krt5rtTA;tetCre:Fgfr1$^{fl/fl}$;Fgfr2$^{fl/fl}$ mice had normal gross histology compared to control. Representative images, scale bars 50$\mu$m.

h) qPCR of Krt5rtTA;tetCre:Fgfr1$^{fl/fl}$;Fgfr2$^{fl/fl}$ glands compared to control showed a trend of reduced but not significant trend for duct markers (n=6 for each phenotype). Unpaired student t-test was used to calculate significance (*p<0.05). Data is presented as mean values +/- SEM and source data are provided as a Source Data file.

i) Quantification of GFP expression in IHC sections at day 0 showed similar levels of expression in control and after *Fgfr1b* and *Fgfr2b* deletion although lower detection was observed in females (n=4 and 3) compared to male glands (n=4 for each genotype). Graph shows mean measurements with SEM and provided as a Source Data file.

## Supplementary Figure 3

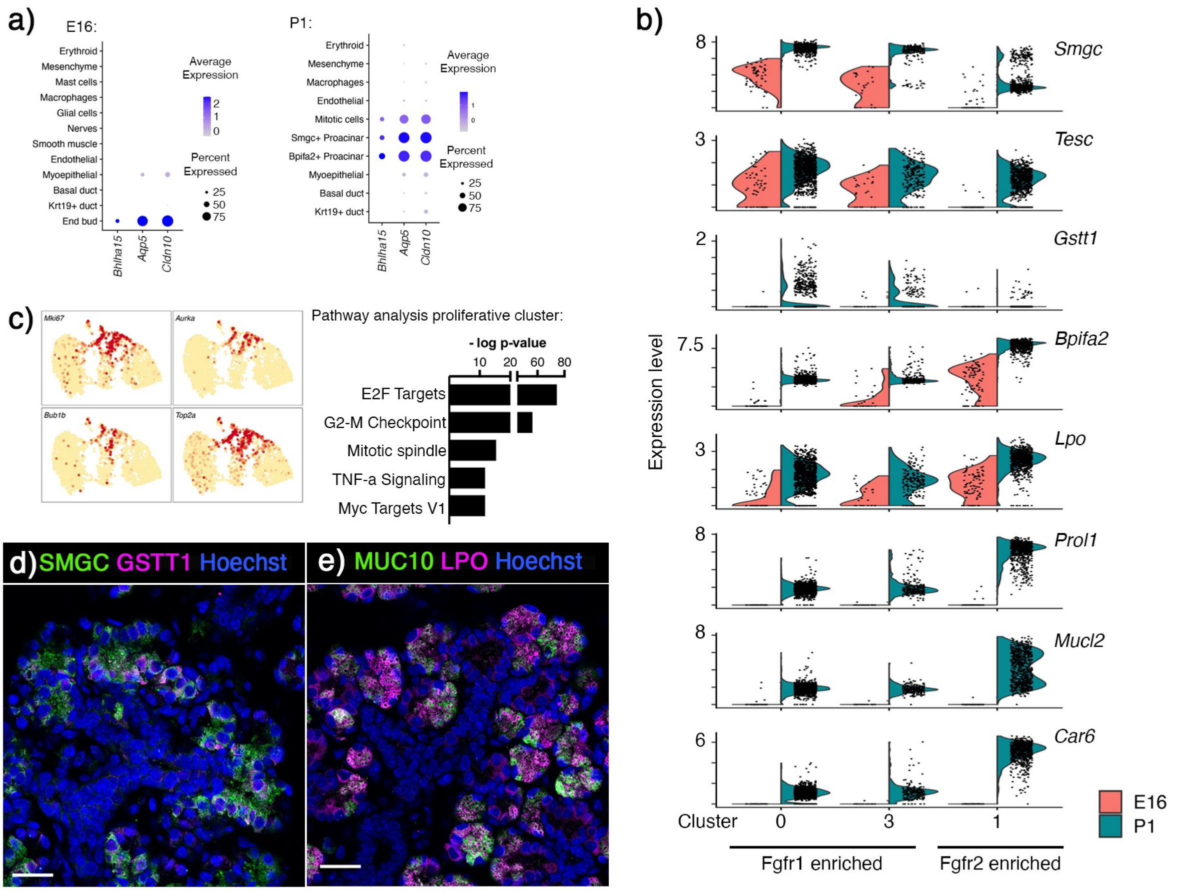


**Supplementary Figure 3: Acinar markers are expressed in both Smgc+ and Bpifa2+ proacinar populations and proliferating acinar cells, related to Figure 4.**

a) Dotplots showing specific enrichment of canonical acinar markers *Aqp5*, *Bhlha15* and *Cldn10* at E16 and P1.

b) Split violin plots showing expression levels of acinar markers at E16 and P1 in Fgfr1 and Fgfr2 enriched clusters.

c) Acinar cluster 2 contains both Bpifa2+ and Smgc+ cells and pathway analysis confirmed these cells were actively proliferating. Proliferation markers, *Mki67*, *Aurka*, *Bub1b* and *Top2a* were among the defining genes and are shown in UMAPs.

d) Representative images showing overlap between SMGC (green) and GSTT1 (magenta) in P1 SMG. Nuclei were stained with Hoechst (blue). Staining repeated on 3 biological replicates showing similar results. Scale bar: 20 $\mu$m.

e) Representative images showing overlap between MUC10 (green) and LPO (magenta) in P1 SMG. Hoechst (nuclei, blue). Staining repeated on 3 biological replicates showing similar results. Scale bar: 20 $\mu$m.

## Supplementary Figure 4

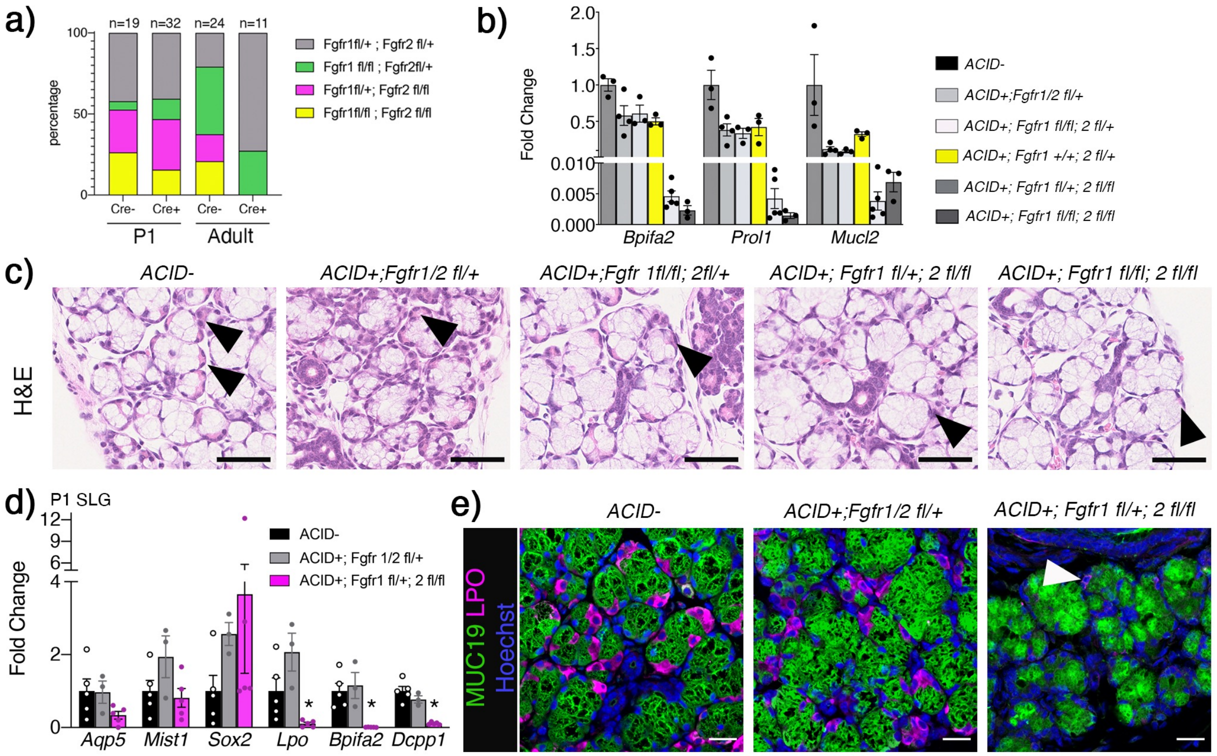


**Supplementary Figure 4: Fgfr2 deletion in SLG acinar cells leads to loss of serous markers, related to Figure 5.**

a) Graph showing distribution of genotypes of ACID-Cre mice at P1 and in adult. No ACID-Cre+;Fgfr2$^{fl/fl}$ could be generated due to a lethal phenotype beyond P1-P3 (P: postnatal day 1).

b) qPCR showing showing the comparison of *Bpifa2*, *Prol1* and *Mucl2* after *Fgfr2* deletion with or without *Fgfr1* deletion. n=3 *ACID-*, *Fgfr1$^{fl/fl}$;2$^{fl/+}$* , *Fgfr1$^{+/+}$;2$^{fl/+}$* and *Fgfr1/2$^{fl/fl}$*, n=5 for *Fgfr1/2$^{fl/+}$* and *Fgfr1f$^{/+}$;2$^{fl/fl}$*. Graph shows mean measurements with SEM and is provided as a Source Data file.

c) Hematoxylin and Eosin (H&E) of SLG ACID showed loss of serous cells (arrowheads) in *Fgfr2$^{fl/fl}$* glands. Representative images, scale bars: 50$\mu$m.

d) qPCR showing loss of serous acinar markers in SLGs after *Fgfr2* deletion. One-way ANOVA with Dunnett's test for multiple comparisons to control. *Lpo* *p= 0.0061, *Bpifa2* *p<0.0001, *Dcpp1* *p<0.0001. ACID- n=5, *Fgfr1/2$^{fl/+}$* n=3, *Fgfr1/2$^{fl/fl}$* n=5. Graph shows mean measurements with SEM and is provided as a Source Data file.

e) IHC showed decreased staining of LPO (magenta) and sustained MUC19 (green) after *Fgfr2* deletion. Arrowhead pointing to low LPO expressing cell. Representative images, staining repeated on 3 biological replicates showing similar results. scale bars: 20 $\mu$m.

# Supplementary Figure 5

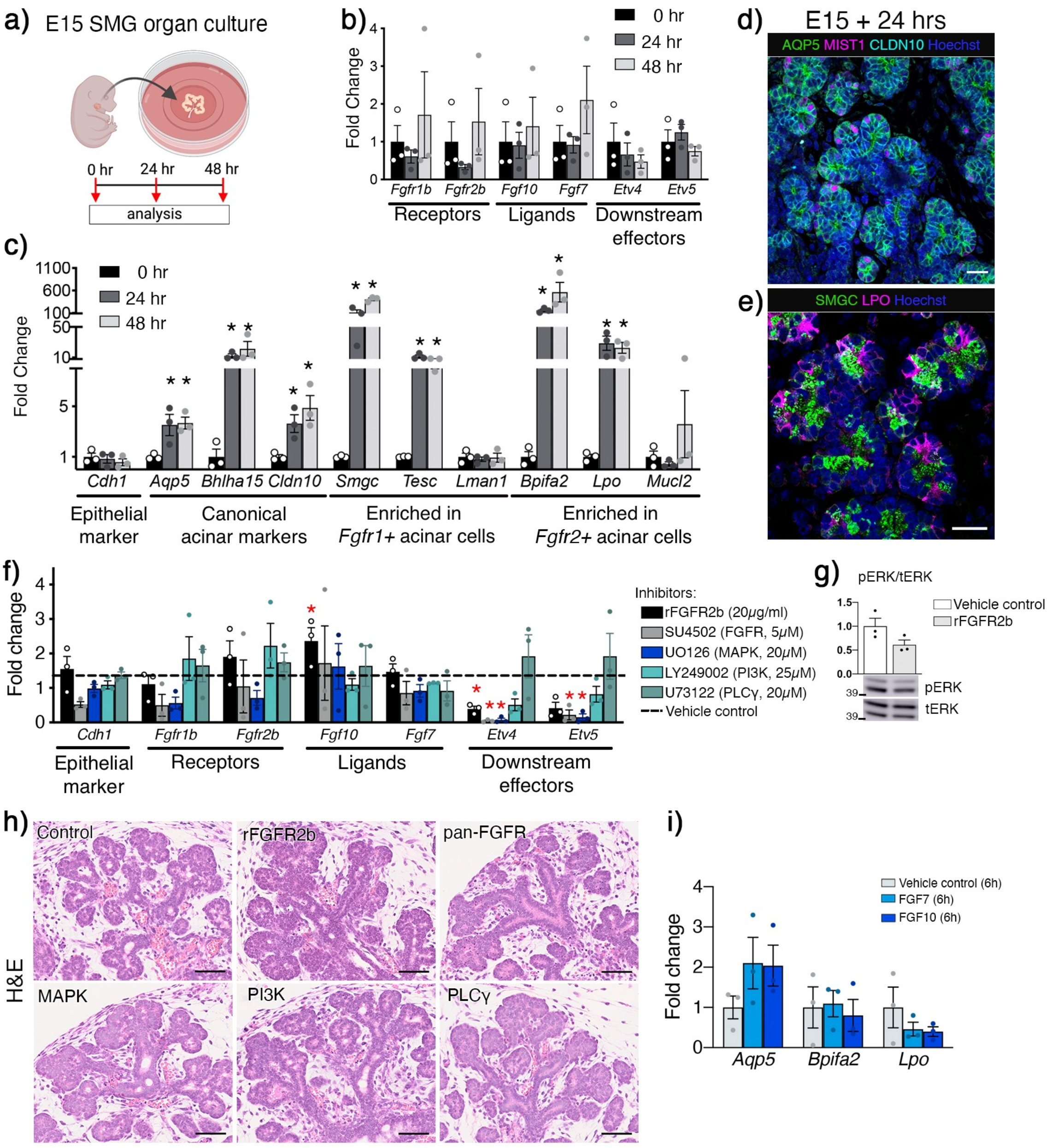

**Supplementary Figure 5: Acinar differentiation in organ culture of E15 SMG reiterates in vivo differentiation, related to Figure 6.**

a) E15 SMGs were cultured for 24 and 48 hrs before analysis. Created with BioRender.com.
b) Downstream FGF signaling pathway within 24 to 48h of organ culture (n=3). One-way ANOVA with Dunnett's test for multiple comparisons to control. Graph shows mean measurements with SEM and provided as a Source Data file.
c) Acinar specific markers detected by qPCR significantly increase within 24 to 48h of organ culture (n=3). One-way ANOVA with Dunnett's test for multiple comparisons to control (*Aqp5* *p=0.0063 and 0.0045, *Bhlha15* *p=0.0077 and 0.0040, *Cldn10* *p=0.005 and 0.0017, *Smgc* *p=0.0003 and <0.0001, *Tesc* *p=<0.0001 for both, *Bpifa2* *p<0.0001 for both, *Lpo* *p=0.0012 and 0.0018). Graph shows mean measurements with SEM and provided as a Source Data file.
d) Acinar markers AQP5 (green), MIST1 (magenta) and CLDN10 (cyan) can be detected in E15 + 24hrs. Representative images, staining repeated on 3 biological replicates showing similar results. scale bar : 50$\mu$m.
e) The two acinar subpopulations can be detected by expression of SMGC (green) and LPO (magenta) in E15 +24hrs. Representative images, staining repeated on 3 biological replicates showing similar results. scale bar: 20$\mu$m.
f) Receptors, ligands and downstream effectors in E15 organ culture after 24 hrs of inhibitor treatments compared to their vehicle control. One-way ANOVA with Dunnett's test for multiple comparisons to control and students t-test when comparing two groups (rFGFR2b). *Fgf10* *p=0.0389, *Etv4* *p=0.0334, 0.0006 and 0.0015, *Etv5* *p=0.047 and 0.0073. n=3 for each treatment group. Graph shows mean measurements with SEM and is provided in the Source Data file.
g) E15 SMGs cultured for 24 hrs with rFGFR2b showed reduction in pERK/tERK compared to vehicle control. Graphs shows quantification of Western Blot (n=3), images are representative bands from the two groups. Full scans of blots are provided in the Source Data file.
h) Hematoxylin and Eosin (H&E) staining showed similar gross histology in all groups after 24hrs + indicated inhibitor treatment. Representative images, scale bars: 50 $\mu$m.
i) E15 SMGs cultured for 6 hrs with FGF7 or FGF10 and is compared to their vehicle control (n=3 for each group). One-way ANOVA with Dunnett's test for multiple comparisons was performed to determine significance. Graph shows mean with SEM and is provided in the Source Data file.

Supplementary Table 1: Resources used in this work.

| REAGENT or RESOURCE | SOURCE | IDENTIFIER |
|---|---|---|
| **Antibodies** | | |
| SOX10 | Santa Cruz Biotechnology | sc-17342 |
| E-Cadherin | BD Biosciences | 610182 |
| GFP | Abcam | ab13970 |
| SMGC | Lifespan Bioscience | LS-C154825 |
| LPO | ThermoFisher Scientific | PA1-46353 |
| GSTT1 | Lifespan Bioscience | LS-B10781 |
| MUC10/PROL1 | Everest | EB10617 |
| Ki67 | BD Pharmigen | 550609 |
| MIST1 | Cell Signaling | 14896 |
| AQP5 | Alomone labs | AQP-005 |
| CLAUDIN10 | ThermoFisher Scientific | 38-8400 |
| Cleaved Caspase3 | Cell Signaling | 9664S |
| P44/42 MAPK (Erk1/2) | Cell Signaling | 9102S |
| Phospho-p44/42 MAPK (Erk1/2) | Cell Signaling | 4370S |
| Alexa Fluor® 488 AffiniPure F(ab')$_2$ Fragment Donkey Anti-Goat IgG (H+L) | Jackson Immunoresearch Laboratories | 705-546-147 |
| Alexa Fluor® 647 AffiniPure F(ab')$_2$ Fragment Donkey Anti-Goat IgG (H+L) | Jackson Immunoresearch Laboratories | 705-606-147 |
| Cy™3 AffiniPure F(ab')$_2$ Fragment Donkey Anti-Rabbit IgG (H+L) | Jackson Immunoresearch Laboratories | 711-166-152 |
| Rabbit IgG VisUCyte HRP Polymer Antibody | R&D Systems | VC003-025 |
| Alexa Fluor™ 488 Tyramide Reagent | ThermoFisher Scientific | B40953 |
| Alexa Fluor™ 594 Tyramide Reagent | ThermoFisher Scientific | B40957 |
| Alexa Fluor™ 647 Tyramide Reagent | ThermoFisher Scientific | B40958 |
| Anti-rabbit, HRP-linked antibody | Cell signaling | 7074 |
| DAPI (Dihydrochloride) | Millipore Sigma | 268298 |
| Hoechst 33342 | ThermoFisher Scientific | H3570 |
| **Chemicals, peptides, and recombinant proteins** | | |
| PBS 1X | Quality Biological, Inc | 114-058-101 |
| DMEM/F12 | ThermoFisher Scientific | 11320033 |
| Penicillin-Streptomycin | ThermoFisher Scientific | 15140122 |
| Transferrin | ThermoFisher Scientific | 11107018 |
| Vitamin C | Sigma Aldrich | A4544 |
| Recombinant mouse FGFR2 beta (IIIb) Fc Chimera protein, CF | R&D Systems | 708-MF-050 |
| SU5402 | Millipore Sigma | 572630 |
| UO126 | Millipore Sigma | 662005 |
| U73122 | Millipore Sigma | 662035 |
| Ly249002 | Biotechne | 1130 |
| Dimethyl sulfoxide DMSO | Sigma Aldrich | 34869 |
| Recombinant human FGF7 protein | R&D Systems | 251-KG-050 |

| | | |
|---|---|---|
| Recombinant human FGF10 protein | R&D Systems | 345-FG-025 |
| Recombinant mouse FGF acidic/FGF1 protein | R&D Systems | 4686-FA-025 |
| Recombinant human FGF basic/FGF2/bFGF protein | R&D Systems | 233-FB-025 |
| Bovine serum albumin solution (BSA) | Sigma Aldrich | A9205 |
| 16% Paraformaldehyde (formaldehyde) Aqueous solution | Electron Microscopy Sciences | 15700 |
| Xylene substitute | Sigma Aldrich | A5597-1GAL |
| Ethanol 100% | Sigma Aldrich | E7023 |
| Ethanol 95% | Sigma Aldrich | 493538 |
| Acetone | Sigma Aldrich | 179973 |
| Methanol | ThermoFisher Scientific | A412-4 |
| Tris Base | Millipore Sigma | 648310-M |
| EDTA | Millipore Sigma | E1161 |
| Tween 20 | Quality Biological, Inc | A611-M147-13 |
| PBS 10X | Quality Biological, Inc | 119-069-101 |
| Boric Acid | Sigma Aldrich | B0394-100G |
| Sodium Chloride | Sigma Aldrich | s3014-500G |
| 4-Iodophenylboronic acid | Sigma Aldrich | 4-471933-5G |
| 30% (w/w) stabilized $H_2O_2$ | Sigma Aldrich | H1009 |
| Normal donkey serum | Jackson Immunoresearch Laboratories | 017-000-121 |
| BLOXALL® Endogenous Peroxidase and Alkaline Phosphatase Blocking Solution | Vector laboratories | SP-6000 |
| Doxycycline (5001C w/6000ppm) | Animal Specialties and Provision | 1813434 |
| Thermo Scientific™ Shandon™ Immu-Mount™ | ThermoFisher Scientific | 9990402 |
| Fluoro-gel II, with DAPI | Electron Microscopy Sciences | 17958-50 |
| Pierce™ RIPA buffer | ThermoFisher Scientific | 89901 |
| Halt™ Protease &Phosphatase Inhibitor Coctail (100x) | ThermoFisher Scientific | 78441 |
| Micro BCA™ protein assay kit | ThermoFisher Scientific | 23235 |
| NuPage™ 4 to12%, Bis-Tris mini protein gel | ThermoFisher Scientific | NPO322BOX |
| iBlot™ 2 Transfer Stacks, PVDF mini | ThermoFisher Scientific | IB24002 |
| NuPage™ LDS Sample Buffer (4x) | ThermoFisher Scientific | NP0007 |
| NuPage™ Sample Reducing Agent (10x) | ThermoFisher Scientific | NP0009 |
| TBS, 10x | Corning | 46-012-CM |
| SuperSignal™ West Dura Chemiluminescent Extended Substrate | ThermoFisher Scientific | 34075 |
| Critical commercial assays | | |
| M.O.M (Mouse on Mouse) Immunodetection Kit | Vector Laboratories | BMK-2202 |
| Superscript III First-Strand Synthesis System | Life Technologies | 18080051 |
| RNAqueous-4PCR kit and DNase removal reagent | ThermoFisher Scientific | AM1914 |
| RNAqueous- PCR micro kit with Dnase treatment | ThermoFisher Scientific | AM1931 |
| iScript cDNA Synthesis Kit | Bio-Rad | 1708890 |
| iQ SYBR Green Supermix | Bio-Rad | 1708882 |
| **Deposited data** | | |
| scRNAseq of murine SMG at multiple developmental stages | [1] | GSE150327 |

| | | | |
|---|---|---|---|
| scRNAseq of human minor salivary gland | | [2] | GSE180544 |
| scRNAseq of human minor salivary gland | | [3] | https://www.covid19cellatlas.org/ |
| scRNAseq of human SMG and PG | | [4] | GSE201333 |
| **Experimental models: Organisms/strains** | | | |
| Timed-pregnant ICR Female Mice | | Envigo | ICR (CD-1®) |
| Crect | | [5] | N/A |
| Krt14Cre | | [6] | N/A |
| Krt5Cre | | [7] | N/A |
| Krt5rtTA; tet-Cre | | [8] | N/A |
| *ACID (*Aqp5 cre knock-in) | | [9] | N/A |
| $Fgfr1^{flox}$ | | [10] | N/A |
| $Fgfr2^{flox}$ | | [11] | N/A |
| $Gt(ROSA)26Sor^{tm4(ACTB\text{-}tdTomato,\text{-}EGFP)Luo}$/J | | The Jackson Laboratory | N/A |
| B6.Cg-$Gt(ROSA)26Sor^{tm9(CAG\text{-}tdTomato)Hze}$/J | | The Jackson Laboratory | N/A |
| **Oligonucleotides** | | | |
| *Fgfr1b F* | AGAGCGGGGAGTATGTGTGTAAGGTTTC | Life Technologies | N/A |
| *Fgfr1b R* | TGGTGACAGTGAGCCACGCAGAC | Life Technologies | N/A |
| *Fgfr2b F* | TGGCTCTGTTCAATGTGACGGAGATGGATG | Life Technologies | N/A |
| *Fgfr2b R* | AGGCGCTTGCTGTTTGGGCAGGAC | Life Technologies | N/A |
| *Krt5 F* | TCCTGTTGAACGCCGCTGAC | Life Technologies | N/A |
| *Krt5 R* | CGGAAGGACACACTGGACTGG | Life Technologies | N/A |
| *Krt14 F* | CCTCATCCTCTCAATTCTCCTCTGGCTCTC | Life Technologies | N/A |
| *Krt14 R* | CTTGGTGCGGATCTGGCGGTTGG | Life Technologies | N/A |
| *Krt7 F* | CGCCGCTGAGTGTGGACATCG | Life Technologies | N/A |
| *Krt7 R* | CTGGCTGCTCTTGGCTGACTTCTG | Life Technologies | N/A |
| *Krt19 F* | GCCACCTACCTTGCTCGGATTG | Life Technologies | N/A |
| *Krt19 R* | GTCTCTGCCAGCGTGCCTTC | Life Technologies | N/A |
| *Cldn3 F* | GCCAACACCATCATCAGGGATTTC | Life Technologies | N/A |
| *Cldn3 R* | GCAGGAGCAACACAGCAAGG | Life Technologies | N/A |
| *Cldn7 F* | GCAGGCGACAACATCATCACAGC | Life Technologies | N/A |
| *Cldn7 R* | GGCAGGGCAAGCACCGAGTC | Life Technologies | N/A |
| *Nkd2 F* | GCCCTGAACCCTCCAGCAAGA | Life Technologies | N/A |
| *Nkd2 R* | CGGATAGCCTTCTGTCAGCCACTC | Life Technologies | N/A |
| *Foxi1 F* | CGAAGGTGGTGGCGAATACG | Life Technologies | N/A |
| *Foxi1 R* | GAAAGGGGCTGGCGTTAGTG | Life Technologies | N/A |
| *Fgf10 F* | TCTTCCTCCTCCTCGTCCTTCTCCTCTCCTTCC | Life Technologies | N/A |
| *Fgf10 R* | CCGCTGACCTTGCCGTTCTTCTCAATCG | Life Technologies | N/A |
| *Fgf7 F* | CAGCCCCGAGCGACACACCAGAAGTTATG | Life Technologies | N/A |
| *Fgf7 R* | TCCTGGGTCCCTTTCACTTTGCCTCGTTTG | Life Technologies | N/A |
| *Etv4 F* | CAGACTTCGCCTACGACTCA | Life Technologies | N/A |
| *Etv4 R* | GCCATAACCCATCACTCCAT | Life Technologies | N/A |
| *Etv5 F* | AAGCCCTTCAAAGTGATAGCGGAGAC | Life Technologies | N/A |
| *Etv5 R* | GTGTCCACAAACTTCCTCTTTCTGTCAATC | Life Technologies | N/A |
| *Cdh1 F* | GACTGGAGTGCCACCACCAAAGAC | Life Technologies | N/A |
| *Cdh1 R* | CGCCTGTGTACCCTCACCATCGG | Life Technologies | N/A |

| | | | |
|---|---|---|---|
| *Aqp5 F* | TCTACTTCTACTTGCTTTTCCCCTCCTC | Life Technologies | N/A |
| *Aqp5 R* | CGATGGTCTTCTTCCGCTCCTCTC | Life Technologies | N/A |
| *Bhlha15 F* | TCGCTGACCGCCACCATACTTAC | Life Technologies | N/A |
| *Bhlha15 R* | CTGCTGCTGCTGCTGCTGTTG | Life Technologies | N/A |
| *Cldn10 F* | CGGGAACCAGCGAGAGCG | Life Technologies | N/A |
| *Cldn10 R* | ATGGAGACTACGAAGGCGACGATT | Life Technologies | N/A |
| *Smgc F* | TCCTCAGCAGTAGACTCCACAGATTC | Life Technologies | N/A |
| *Smgc R* | TGAAAGATCCACCATTATTCTCTCCAACC | Life Technologies | N/A |
| *Tesc F* | CGGATGTTCAGCCCTATGC | Life Technologies | N/A |
| *Tesc R* | GGAAACCTTTATTGTCACCATTAGAGA | Life Technologies | N/A |
| *Lman1 F* | CAAGTGAATGAGGTGAAGAAC | Life Technologies | N/A |
| *Lman1 R* | ATGAAGTGCTGAGTTGTCT | Life Technologies | N/A |
| *Bpifa2 F* | GCTGTCTTCCAACGGCAATGGCATT | Life Technologies | N/A |
| *Bpifa2 R* | CCAATGAAAGGCAGAACCAAGGAGGCTTC | Life Technologies | N/A |
| *Lpo F* | TGACCTTGCTCCAGACTG | Life Technologies | N/A |
| *Lpo R* | CCTTGACCTCTTCCACTGT | Life Technologies | N/A |
| *Mucl2 F* | GTCACCATGAAGTTCCTGGCACTCCTTGT | Life Technologies | N/A |
| *Mucl2 R* | TCACCAGCAGAATCAGCAGTTCCAGAAGTT | Life Technologies | N/A |
| *Gstt1 F* | GCGGCAGTTCACAACTCACAGTTCACAAT | Life Technologies | N/A |
| *Gstt1 R* | TGGAGCTGAGCCTGAGAGCCATCAT | Life Technologies | N/A |
| *Ramp1 F* | GCTACCGCCTAACTCTCATCACAGAATCTC | Life Technologies | N/A |
| *Ramp1 R* | GACAACAGGAACACAGAGGACCATCAGA | Life Technologies | N/A |
| *Cdkn1c F* | CGACTGAGAGCAAGCGAACA | Life Technologies | N/A |
| *Cdkn1c R* | TTCTGGCTGATTGGTGATGGA | Life Technologies | N/A |
| *Prol1 F* | ACC ACA CCA GCA ACA ACC ACA A | Life Technologies | N/A |
| *Prol1 R* | TGG CTG TAG AGG TGC TAG GCT TAG | Life Technologies | N/A |
| *Dcpp1 F* | GGGTATTATGTGGGCAATTCTGAGT | Life Technologies | N/A |
| *Dcpp1 R* | TCACACATGAACTGTTTTCACTTCC | Life Technologies | N/A |
| *Elf5 F* | CGTCAGCGTGTTCAGTTTATTGATTCTC | Life Technologies | N/A |
| *Elf5 R* | GTTCGGCTATCGTGGCTTGTTAGG | Life Technologies | N/A |
| *Sox2 F* | CAGCATGTCCTACTCGCAGCAG | Life Technologies | N/A |
| *Sox2 R* | TGGAGTGGGAGGAAGAGGTAACC | Life Technologies | N/A |
| *mKi67 F* | TTGCCTCCTAATACACCACTGA | Life Technologies | N/A |
| *mKi67 R* | CCGTTCCTTGATGATTGTCTTGA | Life Technologies | N/A |
| *Acta2 F* | GCATGGATGGCATCAATCAC | Life Technologies | N/A |
| *Acta2 R* | ACCTATCTGGTCACCTGTATGTA | Life Technologies | N/A |
| *Cnn1 F* | CGCACAACTACTACAACTC | Life Technologies | N/A |
| *Cnn1 R* | CCCAAACCGTAACCCTATA | Life Technologies | N/A |
| *Fgf2 F* | GCGAGAAGAGCGACCCACAC | Life Technologies | N/A |
| *Fgf2 R* | GAAGCCAGCAGCCGTCCATC | Life Technologies | N/A |
| *Pdgfra F* | GAGGACTTGGGTGATGTGGAGAGAG | Life Technologies | N/A |
| *Pdgfra R* | ACGCCGCTGAGATGCTACTGAC | Life Technologies | N/A |
| *Col1a1 F* | TGGTCCTGCTGGTCCTGCTGGTC | Life Technologies | N/A |
| *Col1a1 R* | GCCTCTGTCACCTTGTTCGCCTGTC | Life Technologies | N/A |
| *Rps29 F* | GGAGTCACCCACGGAAGTTCGG | Life Technologies | N/A |
| *Rps29 R* | GGAAGCACTGGCGGCACATG | Life Technologies | N/A |
| *KRT14-Cre F* | TTCCTCAGGAGTGTCTTCGC | Life Technologies | N/A |

| | | | |
|---|---|---|---|
| *KRT14-Cre R* | GTCCATGTCCTTCCTGAAGC | Life Technologies | N/A |
| *ACID F* | TGCCCAAGAAGAAGAGGAAGGTGT | Life Technologies | N/A |
| *ACID R* | GCCGCATAACCAGTGAAACAGCAT | Life Technologies | N/A |
| *Cre F* | GCGGTCTGGCAGTAAAAACTATC | Life Technologies | N/A |
| *Cre R* | GTGAAACAGCATTGCTGTCACTT | Life Technologies | N/A |
| *tetCre F* | GTCGATGCAACGAGTGATGAG | Life Technologies | N/A |
| *tetCre R* | GAACCTGGTCGAAATCAGTGC | Life Technologies | N/A |
| *Krt5rtTA F* | CCGGATCCACCATGCCTAAGAGCCCACGACCGTCTAGATTAGATAAAAGT | Life Technologies | N/A |
| *Krt5rtTA R* | ATCTGAATGTACTTTTGCTCCATTGCGATACT | Life Technologies | N/A |
| *Fgfr1 WT F* | CCAGTAACTGTACCAATGAGCTGTAAGCAT | Life Technologies | N/A |
| *Fgfr1 WT R* | TGCCCACCATGCTCCTGCTTCCTTCAGAGC | Life Technologies | N/A |
| *Fgfr1 flox F* | ACCTCAGGAACCTCGAATAAGCCACCATCC | Life Technologies | N/A |
| *Fgfr1 flox R* | AGGTTCCCTCCTCTTGGATGACTTTAG | Life Technologies | N/A |
| *Fgfr2 WT F* | TTCCTGTTCGACTATAGGAGCAACAGGCGG | Life Technologies | N/A |
| *Fgfr2 WT R* | GAGAGCAGGGTGCAAGAGGCGACCAGTCAG | Life Technologies | N/A |
| *Fgfr2 flox F* | TTCCTGTTCGACTATAGGAGCAACAGGCGG | Life Technologies | N/A |
| *Fgfr2 flox R* | CATAGCACAGGCCAGGTTGTTCATTTCCAT | Life Technologies | N/A |
| *mTmG common* | CTCTGCTGCCTCCTGGCTTCT | Life Technologies | N/A |
| *mTmG WT* | CGAGGCGGATCACAAGCAATA | Life Technologies | N/A |
| *mTmG Mutant* | TCAATGGGCGGGGGTCGT T | Life Technologies | N/A |
| *tdTomato WT F* | AAGGGAGCTGCAGTGGAGTA | Life Technologies | N/A |
| *tdTomato WT R* | CCGAAAATCTGTGGGAAGTC | Life Technologies | N/A |
| *tdTomato Mut F* | CTGTTCCTGTACGGCATGG | Life Technologies | N/A |
| *tdTomato Mut R* | GGCATTAAAGCAGCGTATCC | Life Technologies | N/A |
| RNA probe for *Fgfr1* | | ACDBio | N/A |
| RNA probe for *Fgfr2* | | ACDBio | N/A |
| RNA probe for *Bhlha15* | | ACDBio | N/A |
| RNA probe for *Krt5* | | ACDBio | N/A |
| RNA probe for *Fgf7* | | ACDBio | N/A |
| RNA probe for *Cnn1* | | ACDBio | N/A |
| RNA probe for *Pdgfra* | | ACDBio | N/A |
| **Software and algorithms** | | | |
| R & R studio | | https://rstudio.com/ | N/A |
| Seurat V4 | | [12] | N/A |
| FIJI (2.14.0/1.54f) | | [13] | N/A |
| Bio-Rad CFX Maestro 2.3 | | Bio-Rad | N/A |
| Prism 9 | | Graphpad | N/A |

| | | |
|---|---|---|
| Photoshop 2024 | Adobe | N/A |
| Excel (16.77.1) | Microsoft | N/A |
| Word (16.77.1) | Microsoft | N/A |
| Endnote 20 | Clarivate | N/A |
| Confocal NIS-Elements Package | Nikon | N/A |
| NDP.view2 | Hamamatsu | U12388-01 |
| Beacon Designer 8 | PREMIER Biosoft | N/A |
| **Other** | | |
| Whatman Nuclepore Track-etch filters (13 mm, 0.1 µm pore size) | VWR | WHA110405 |
| 50 mm Dish, No. 1.5 Coverslip, 14 mm Glass Diameter, Uncoated | MatTek | P50G-1.5-14-F |
| Tissue-Tek® OCT™ compound | Sakura/VWR | 25608-930 |


1. Hauser, B.R., Aure, M.H., Kelly, M.C., Hoffman, M.P., Chibly, A.M., and Genomics Computational Biol, C. (2020). Generation of a Single-Cell RNAseq Atlas of Murine Salivary Gland Development. iScience *23*, 35, 101838. 10.1016/j.isci.2020.101838.
2. Costa-da-Silva, A.C., Aure, M.H., Dodge, J., Martin, D., Dhamala, S., Cho, M., Rose, J.J., Bassim, C.W., Ambatipudi, K., Hakim, F.T., et al. (2022). Salivary ZG16B expression loss follows exocrine gland dysfunction related to oral chronic graft-versus-host disease. iScience *25*, 103592. 10.1016/j.isci.2021.103592.
3. Huang, N., Pérez, P., Kato, T., Mikami, Y., Okuda, K., Gilmore, R.C., Conde, C.D., Gasmi, B., Stein, S., Beach, M., et al. (2021). SARS-CoV-2 infection of the oral cavity and saliva. Nat Med *27*, 892-903. 10.1038/s41591-021-01296-8.
4. Jones, R.C., Karkanias, J., Krasnow, M.A., Pisco, A.O., Quake, S.R., Salzman, J., Yosef, N., Bulthaup, B., Brown, P., Harper, W., et al. (2022). The Tabula Sapiens: A multiple-organ, single-cell transcriptomic atlas of humans. Science *376*, eabl4896. 10.1126/science.abl4896.
5. Harlow, D.E., Yang, H., Williams, T., and Barlow, L.A. (2011). Epibranchial Placode-Derived Neurons Produce BDNF Required for Early Sensory Neuron Development. Dev. Dyn. *240*, 309-323. 10.1002/dvdy.22527.
6. Vasioukhin, V., Degenstein, L., Wise, B., and Fuchs, E. (1999). The magical touch: Genome targeting in epidermal stem cells induced by tamoxifen application to mouse skin. Proceedings of the National Academy of Sciences of the United States of America *96*, 8551-8556. 10.1073/pnas.96.15.8551.
7. Ramirez, A., Page, A., Gandarillas, A., Zanet, J., Pibre, S., Vidal, M., Tusell, L., Genesca, A., Whitaker, D.A., Melton, D.W., and Jorcano, J.L. (2004). A keratin K5Cre transgenic line appropriate for tissue-specific or generalized Cre-mediated recombination. Genesis *39*, 52-57. 10.1002/gene.20025.
8. Vitale-Cross, L., Amornphimoltham, P., Fisher, G., Molinolo, A.A., and Gutkind, J.S. (2004). Conditional expression of K-ras in an epithelial compartment that includes the stem cells is sufficient to promote squamous cell carcinogenesis. Cancer Res. *64*, 8804-8807. 10.1158/0008-5472.Can-04-2623.
9. Flodby, P., Borok, Z., Banfalvi, A., Zhou, B., Gao, D., Minoo, P., Ann, D.K., Morrisey, E.E., and Crandall, E.D. (2010). Directed expression of Cre in alveolar epithelial type 1

cells. American journal of respiratory cell and molecular biology *43*, 173-178. 10.1165/rcmb.2009-0226OC.
10. Trokovic, R., Trokovic, N., Hernesniemi, S., Pirvola, U., Weisenhorn, D.M.V., Rossant, J., McMahon, A.P., Wurst, W., and Partanen, J. (2003). FGFR1 is independently required in both developing mid- and hindbrain for sustained response to isthmic signals. Embo Journal *22*, 1811-1823. 10.1093/emboj/cdg169.
11. Yu, K., Xu, J.S., Liu, Z.H., Sosic, D., Shao, J.S., Olson, E.N., Towler, D.A., and Ornitz, D.M. (2003). Conditional inactivation of FGF receptor 2 reveals an essential role for FGF signaling in the regulation of osteoblast function and bone growth. Development *130*, 3063-3074. 10.1242/dev.00491.
12. Stuart, T., Butler, A., Hoffman, P., Hafemeister, C., Papalexi, E., Mauck, W.M., 3rd, Hao, Y., Stoeckius, M., Smibert, P., and Satija, R. (2019). Comprehensive Integration of Single-Cell Data. Cell *177*, 1888-1902.e1821. 10.1016/j.cell.2019.05.031.
13. Schindelin, J., Arganda-Carreras, I., Frise, E., Kaynig, V., Longair, M., Pietzsch, T., Preibisch, S., Rueden, C., Saalfeld, S., Schmid, B., et al. (2012). Fiji: an open-source platform for biological-image analysis. Nature Methods *9*, 676-682. 10.1038/nmeth.2019.
