# Supplementary material for: FGFR2 is essential for salivary gland duct homeostasis and MAPK-dependent seromucous acinar cell differentiation
Source: Nat Commun. 2023 Oct 14;14:6485. doi: 10.1038/s41467-023-42243-0 (PMC10576811; doi:10.1038/s41467-023-42243-0)
Supplement: Supplementary file 3 — Reporting Summary [file 41467_2023_42243_MOESM3_ESM.pdf]

nature portfolio

Corresponding author(s): Marit H. Aure

Last updated by author(s): Sep 28, 2023

Reporting Summary

Nature Portfolio wishes to improve the reproducibility of the work that we publish. This form provides structure for consistency and transparency in reporting. For further information on Nature Portfolio policies, see our Editorial Policies and the Editorial Policy Checklist.

Statistics

For all statistical analyses, confirm that the following items are present in the figure legend, table legend, main text, or Methods section.

n/a	Confirmed	
☐	☒	The exact sample size (*n*) for each experimental group/condition, given as a discrete number and unit of measurement
☐	☒	A statement on whether measurements were taken from distinct samples or whether the same sample was measured repeatedly
☐	☒	The statistical test(s) used AND whether they are one- or two-sided<br>*Only common tests should be described solely by name; describe more complex techniques in the Methods section.*
☒	☐	A description of all covariates tested
☒	☐	A description of any assumptions or corrections, such as tests of normality and adjustment for multiple comparisons
☐	☒	A full description of the statistical parameters including central tendency (e.g. means) or other basic estimates (e.g. regression coefficient) AND variation (e.g. standard deviation) or associated estimates of uncertainty (e.g. confidence intervals)
☐	☒	For null hypothesis testing, the test statistic (e.g. *F*, *t*, *r*) with confidence intervals, effect sizes, degrees of freedom and *P* value noted<br>*Give P values as exact values whenever suitable.*
☒	☐	For Bayesian analysis, information on the choice of priors and Markov chain Monte Carlo settings
☒	☐	For hierarchical and complex designs, identification of the appropriate level for tests and full reporting of outcomes
☒	☐	Estimates of effect sizes (e.g. Cohen's *d*, Pearson's *r*), indicating how they were calculated

Our web collection on statistics for biologists contains articles on many of the points above.

Software and code

Policy information about availability of computer code

Data collection	qPCR results were collected using a C1000 Touch Thermal Cycler and CFX96 Real-Time System connected with the Bio-Rad CFX Maestro 2.3 Software (All from Bio-Rad).<br>All confocal images of IHC stains were captured using a Nikon A1R+ MP microscope (40x or 60x oil objectives) and the Confocal NIS-Elements Package (Nikon instruments) while in situ images were taken using a Dragonfly Spinning Disk system (60x objective).<br>Slides with H&E stained slides were scanned using a S60 NanoZoomer Digital (Hamamatsu).<br>Wester blots were imaged on a GE Amersham Imager AI680 using automatic exposure settings.
Data analysis	IHC and Western Blot quantification was done using FIJI and further analysis and calculations were done using Microsoft Excel and Prims 9.<br>Images from the S60 Nanozoomer Digital slide scanner was exported using the NDP.view 2 software (Hamamatsu).<br>Analysis of qPCR results were done using Microsoft excel and Prism9.<br>All scRNAseq data was analyzed using R and R studio (https://rstudio.com/) and Seurat V4. No custom functions or code was used for the analysis in this work.

For manuscripts utilizing custom algorithms or software that are central to the research but not yet described in published literature, software must be made available to editors and reviewers. We strongly encourage code deposition in a community repository (e.g. GitHub). See the Nature Portfolio guidelines for submitting code & software for further information.

Data

Policy information about availability of data

All manuscripts must include a data availability statement. This statement should provide the following information, where applicable:

- Accession codes, unique identifiers, or web links for publicly available datasets
- A description of any restrictions on data availability
- For clinical datasets or third party data, please ensure that the statement adheres to our policy

All scRNAseq used in this study are from previously deposited data. Murine SMG at multiple developmental stages is from GSE150327 [https://www.ncbi.nlm.nih.gov/geo/query/acc.cgi?acc=GSE150327]30. Human minor salivary glands scRNAseq datasets are from GSE180544 [https://www.ncbi.nlm.nih.gov/geo/query/acc.cgi?acc=GSE180544]32 and through a website portal https://www.covid19cellatlas.org/ 33, while human SMG and PG scRNAseq is from GSE201333 [https://www.ncbi.nlm.nih.gov/geo/query/acc.cgi?acc=GSE201333]31.
Specific values used to generate graphs in this paper are provided in the Source data file.

Field-specific reporting

Please select the one below that is the best fit for your research. If you are not sure, read the appropriate sections before making your selection.

☒ Life sciences ☐ Behavioural & social sciences ☐ Ecological, evolutionary & environmental sciences

For a reference copy of the document with all sections, see nature.com/documents/nr-reporting-summary-flat.pdf

Life sciences study design

All studies must disclose on these points even when the disclosure is negative.

Sample size	Sample size for each experiment was determined based on our previous work and the number of specific genotypes we could logistically generate. We included a minimum of 3 biological replicates for each group/treatment/timepoint/sex.
Data exclusions	No data was excluded from analysis.
Replication	Organ culture experiments were replicated using embryos from 3 independent litters for each treatment group.<br>IHC staining and qPCR was repeated on 3-4 biological replicates fro each genotype/treatment/sex.
Randomization	For organ culture experiments, two to three glands from separate embryos were placed per filter/dish and randomly selected for the various treatments. Each treatment was replicated using embryos from 3 independent litters.<br>Experiments with embryonic or neonatal glands from Cre strains, data was collected and then organized based on genotyping results.<br>Experiments using adult mice with specific genotypes, animals were used based on genotyping results and at least 3 animals were used per group/treatment/timepoint/sex.
Blinding	Samples used for IHC were picked based on genotyping results and all quantification of histological images were performed blinded.

Reporting for specific materials, systems and methods

We require information from authors about some types of materials, experimental systems and methods used in many studies. Here, indicate whether each material, system or method listed is relevant to your study. If you are not sure if a list item applies to your research, read the appropriate section before selecting a response.

Materials & experimental systems

n/a	Involved in the study	
☐	☒	Antibodies
☒	☐	Eukaryotic cell lines
☒	☐	Palaeontology and archaeology
☐	☒	Animals and other organisms
☒	☐	Human research participants
☒	☐	Clinical data
☒	☐	Dual use research of concern

Methods

n/a	Involved in the study	
☒	☐	ChIP-seq
☒	☐	Flow cytometry
☒	☐	MRI-based neuroimaging

Antibodies

Antibodies used	SOX10 (1:100, Santa Cruz Biotechnology, sc-17342), E-cadherin (1:200, BD Biosciences, #610182), GFP (1:500, Abcam ab13970),

Antibodies used	SMGC (1:200, Lifespan Bioscience, LS-C154825), LPO (1:200, Thermo Fisher Scientific, PA1-46353), GSTT1 (1:100, Lifespan Bioscience # LS-B10781), MUC10/PROL1 (1:200, Everest, EB10617), Ki67 (1:200, BD Pharmigen, 550609) and Cleaved Caspase 3(1:100, Cell Signaling, #9664S), MIST1 (1:500, Cell Signaling, #14896), AQP5 (1:700, Alomone labs, AQP-005) and CLAUDIN10 (1:1000, Thermo Fisher Scientific, #38-8400). Secondary antibodies used were Alexa Fluor® 488 AffiniPure F(ab')$_2$ Fragment Donkey Anti-Goat IgG (H +L) (1:250, #705-546-147), Alexa Fluor® 647 AffiniPure F(ab')$_2$ Fragment Donkey Anti-Goat IgG (H+L) (1:250, #705-606-147), Cy™3 AffiniPure F(ab')$_2$ Fragment Donkey Anti-Rabbit IgG (H+L) (1:250, #711-166-152) all from Jackson Immunoresearch Laboratories and Rabbit IgG VisUCyte HRP Polymer Antibody (R&D Systems, VC003-025).
Validation	All antibodies used in this study was purchased from vendors and validation for their applicability can be found at vendors websites. The antibodies have also been confirmed by others and used in multiple publications. We also optimized protocols and dilutions and performed no antibody controls for these antibodies.<br>SOX10 (Santa Cruz Biotechnology, sc-17342) has been validated by vendors and used in several publications including Lombaert et.al. 2013.<br>E-cadherin (BD Biosciences, #610182) has been tested by the company and used in several publications including Miyoshi et.al. 2001.<br>GFP (Abcam ab13970) has been validated by the vendor and customers and used in several peer-reviewed publications including Berg et.al. 2023.<br>SMGC (Lifespan Bioscience LS-C154825) has been validated by the vendor and also used in a peer-reviewed publication, Hauser et.al. 2020.<br>GSTT1 (Lifespan Bioscience LS-B10781) has been validated by the vendor and also used in a peer-reviewed publication, Hauser etl.al. 2020.<br>MUC10/PROL1 (Everest, EB10617) has been validated by the vendor and customers and used in several peer-reviewed publications including Tanaka et.al 2018.<br>Ki67 (BD Pharmigen, 550609) has been validated by the vendor and customers and used in several peer-reviewed publications including Benson et.al 2009.<br>Cleaved Caspase 3(Cell Signaling, #9664S) has been validated by the vendor and customers and used in several peer-reviewed publications including Lv et.al 2023.<br>MIST1 (Cell Signaling, #14896)has been validated by the vendor and customers and used in several peer-reviewed publications including Zhu et al 2022.<br>AQP5 (Alomone labs, AQP-005)has been validated by the vendor and used in several peer-reviewed publications including Larsen et.al 2011.<br>CLAUDIN10 (1Thermo Fisher Scientific, #38-8400) has been validated by the vendor and used in peer-reviewed publications including Tran et.al 2022.<br>phospho-p44/42 MAPK (#4370S Cell Signaling) has been validated by the vendor and used in peer-reviewed publications including Myllymäki et.al. 2023.<br>p44/42 MAPK (#9102 Cell Signaling) has been validated by the vendor and used in peer-reviewed publications including Ballester-Rosado 2022.

Animals and other organisms

Policy information about studies involving animals; ARRIVE guidelines recommended for reporting animal research

Laboratory animals	Strain used:<br>Crect (Harlow et al 2011),<br>Krt14Cre (Vasioukhin et.al 1999),<br>Krt5Cre (Ramirez et al 2004),<br>Krt5rtTA;tet-Cre (Vitale-Cross et al 2004),<br>ACID (Flodby et al 2010),<br>Fgfr1fl/fl (Trocovic et al 2003),<br>Fgfr2fl/fl (Yu et al 2003),<br>mTmG (Gt(ROSA)26Sortm4(ACTB-tdTomato,-EGFP)Luo/J, The Jackson Laboratory) ,<br>tdTomato (B6.Cg-Gt(ROSA)26Sortm9(CAG-tdTomato)Hze/J, The Jackson Laboratory),<br>Timed pregnant ICR (CD-1) females (Envigo).<br>Due to the known sexual dimorphism in adult mouse SMGs, differing outcomes between sexes was considered when using adult mice and findings in this work apply to both sexes.<br>Sex was not considered in experiments using embryonic or neonatal glands since no known sexual dimorphism is present during development.<br>Adult mice used were 6-8 weeks old at the start of experiments.<br>Glands at various development stages were E12, E13,E15 or P1 as indicated for specific experiments/Cre stains.<br><br>Mice were kept in a 14hr on/10hr off light/dark cycle at 74-78F with 50-70% humidity.
Wild animals	This study did not use wild animals.
Field-collected samples	No field-collected samples.
Ethics oversight	All experiments were approved by the NIH Animal Care and Use Committee.

Note that full information on the approval of the study protocol must also be provided in the manuscript.
